# Development of a Robotic Surgery Training System

**DOI:** 10.3389/frobt.2021.773830

**Published:** 2022-01-31

**Authors:** Robin Julia Trute, Carlos Suárez Zapico, Andreas Christou, Daniel Layeghi, Stewart Craig, Mustafa Suphi Erden

**Affiliations:** ^1^ School of Engineering and Physical Sciences, Heriot-Watt University, Edinburgh, United Kingdom; ^2^ School of Informatics, University of Edinburgh, Edinburgh, United Kingdom; ^3^ Edinburgh Centre for Robotics, University of Edinburgh, Edinburgh, United Kingdom

**Keywords:** minimally-invasive surgery, haptic feedback, 3D vision, stereo vision, laparoscopic skill development, robotic training

## Abstract

Robotic Surgery is getting widely spread and applied to more and more clinical cases due to its advantages compared to open surgery, for both the patients and surgeons. However, Robotic Surgery requires a different set of skills and learning compared to open and also laparoscopic surgery. Tele-operation for a robotic system with hand controllers, the delay in the hand commands to be translated into robotic movements, slowness of the robotic movements, remote 2D or 3D vision of the actual operation, and lack of haptic feedback are some of the challenges that Robotic Surgery poses. Surgeons need to go through an intensive training for Robotic Surgery, and the learning and skill development continues throughout their early professional years. Despite the importance of training for Robotic Surgery, there are not yet dedicated, low-cost, and widespread training platforms; rather, surgeons mostly train with the same Robotic Surgery system they use in surgery; hence institutions need to invest on a separate surgical setup for training purposes. This is expensive for the institutions, it provides very limited access to the surgeons for training, and very limited, if any, access to researchers for experimentation. To address these, we have developed in our laboratory a low-cost, and experimental Robotic Surgery Trainer. This setup replicates the challenges that a Robotic Surgery system poses and further provides widespread access through internet connected control of the actual physical system. The overall system is composed of equipment that a standard engineering laboratory can afford. In this paper, we introduce the Robotic Surgery Training System and explain its development, parts, and functionality.

## 1 Introduction

With the advancement of technology, robotic surgery has rapidly become a widely available form of surgical operation (Sheetz et al., 2020). As a result, fully trained surgeons have become much more in demand. However, in many cases, surgical robots are very expensive, and sparsely located making access to such systems very limited for training purposes. In contrast to the high price of Robotic Surgery systems that are usually purchased on an institutional level for training purposes, we present the development and parts of a low cost Robotic Surgery Training System, for both training, and also laboratory based research purposes. Our Robotic Surgery Trainer enables teleoperation of two forceps to perform any standard training game in a laparoscopy training box, 3D vision of the operative field inside the training box (as well as 2D vision on a monitor), haptic feedback to the user, and remote teleoperation of the system through internet using a standard laptop keyboard with 2D vision. Such a system would provide the trainees an easy and widespread access to training for teleoperation with a physical experimental robotic surgery setup. Moreover, this setup is also useful to research various aspects of robotic surgery, ranging from development and testing of tools and control technologies to development and testing of skill training and assessment techniques, and to pilot verification of novel ideas in robotic surgery such as usefulness of haptic feedback.

**FIGURE 1:**
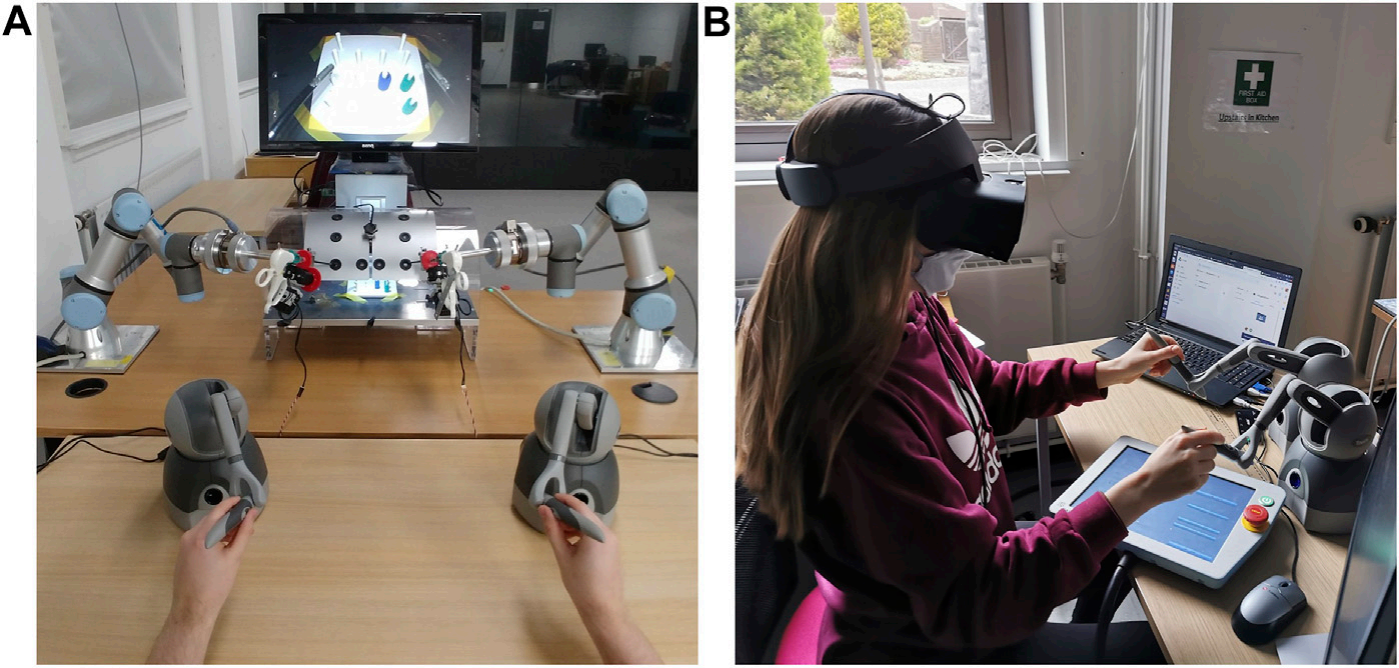
**(A)** The setup of the robotic training system with all components in on-site control mode with 2D vision on-screen modality. **(B)** VR headset setup for 3D vision modality.

**FIGURE 2:**
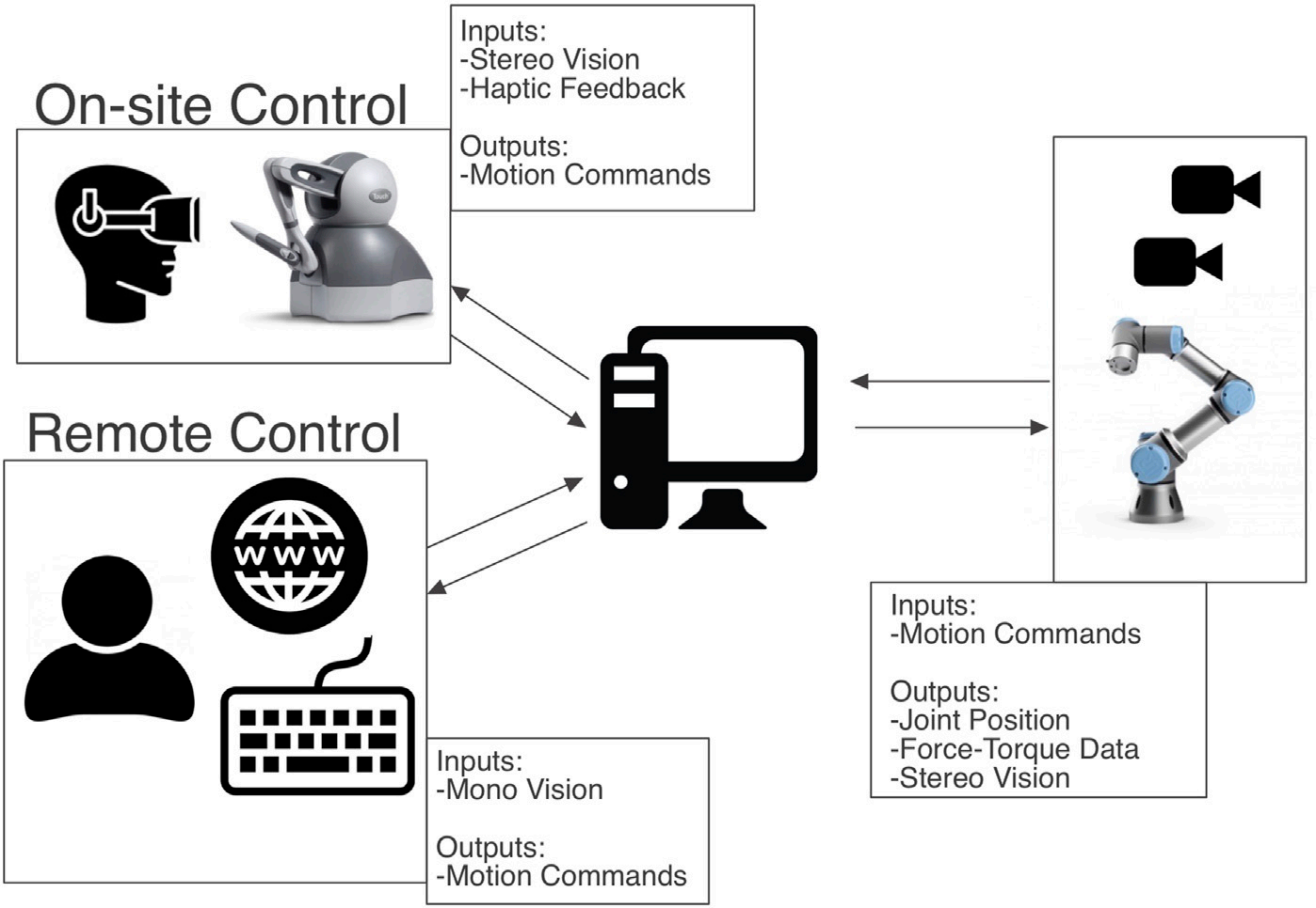
Overall system architecture diagram.

The robotic surgery training system we are presenting here, has multiple use modes which are: on-site operation with haptic feedback through control via haptic devices^1^ and remote control of the system via internet connection and keyboard input control. The first mode can either be used with 2D vision on a screen that is mounted above the training box (see Figure 1A) or with 3D vision via the Oculus Rift S VR headset stereo vision setup (see Figure 1B). Figure 2 shows a general architecture diagram for the two teleoperation modes and the data communication between the user, the main PC and the robot manipulators.

### 1.1 Background

Even though robotic surgery offers many advantages over traditional open surgery, surgeons are faced with increased cognitive load during robotic surgery (Vajsbaher et al., 2020). There are visuo-spatial challenges for trainees that make the learning process difficult (Perkins et al., 2002). Another challenge that trainees face is the time constraint to using real robotic systems like the Da Vinci robot for training. These high-cost systems are needed for surgery and frequently used in hospitals by advanced surgeons. This makes it difficult for students to gain experience with robotic surgery systems. To tackle these challenges our low-cost training system for robotic surgery was developed.

Often, novice surgeons start their training on virtual reality simulators such as the Robotics Surgical Simulator (ROSS)^2^ or the DV-Trainer^3^ (Fisher et al., 2015). The limitation with these setups is that when it comes to simulating flexible visco-elastic materials such as body tissues and their physical interactions with tools, there is still a big simulation to reality gap. An advantage of these training setups is that they can be used to transfer acquired skills from the recorded sessions of experienced surgeons and use them through replaying them to guide the novice surgeons and accelerate their learning curve (Abdelaal et al., 2019). While these virtual systems are useful for surgical training, our system is an actual physical system which can better emulate the physical interaction in and with a real robotic surgery environment.

There are other physical systems that are meant to be solutions for being less expensive than the real robotic surgery systems. These are for example the Raven II (Hannaford et al., 2013) and an Open-Source Research Kit (Kazanzides et al., 2014) for the da Vinci Surgical System^4^. These systems however are mostly for technical research purposes, i.e., for exploring new control algorithms or researching new instrument designs. Whereas the system that we are presenting here is mainly focused on training/surgical education. Both of these other systems are emphasizing the importance of low-cost solutions for robotic surgery platforms to facilitate robotic surgery research; therefore they are in support of the motivation for our system introduced in this paper. While the main aim of the other two research kits mentioned above is “to accelerate progress in surgical robotics research”, i.e., exploring new control algorithms or researching new instrument designs, our system’s main focus is to facilitate robotic surgery training and research for training purposes, though our system does not exclude research for robotic surgery system development.

It is also worth mentioning that our design solution is notably less expensive. While the Raven II is valued at $300,000^5^, the cost of our system is approximately £40,000, which makes it more accessible for educational, and research institutions. We could not find a number for the cost of the Open-Source Research Kit from (Kazanzides et al., 2014), but since the system’s hardware is built from retired da Vinci parts, we assume that it is more expensive than the off-the-shelf components our system is built with. Furthermore, we believe that this Open-Source Research Kit is also less deployable than our system because we assume that there will be a very limited number of systems available because of the limited number of retired da Vinci components. Widespread deployability is an important aspect for our system, since the main goal is an increased accessibility to surgical trainees.

The 2D setup mode of this training system poses the same visual challenges to the surgeons as classic laparoscopy. In laparoscopic minimallay-invasive surgery (MIS), there is non-binocular disparity, and which means that the insufficient depth cues from the MIS environment could cause surgeons to misjudge spatial depth (Bogdanova et al., 2016). Current literature emphasizes the usefulness of 3D vision in robotic surgery systems and classic laparoscopy (Wilhelm et al., 2014). We integrated 3D vision alongside 2D vision to further explore the benefits of these two modalities with respect to trainee learning outcomes.

The goal of this study has been to setup a robotic surgery training system that would make training with a tele-operated physical robotic system accessible to surgeons in training. This involved setting up a training environment that is both low-cost and can also be accessed remotely by anyone through the internet. Therefore, our system also features the modality to be remotely controlled through internet access.

## 2 Materials and Methods

### 2.1 Hardware

The Robotic Surgery Trainer is composed of a laparoscopy training box, two UR3 robot arms, two ATI force/torque sensors, two standard forceps used in laparoscopy, servo motors to control the jaws of the forceps, an Arduino processors to control the servos, two Touch haptic devices, a laptop to control the overall system, two Sony cameras and an Oculus Gaming Headset for 3D vision, and two separate software, one for on-site haptic control of the Robotic Surgery Trainer and another for off-site control through internet connection. The overall system architecture can be seen in Figure 2.

#### 2.1.1 Manipulators

Two UR3 robot arms are used to move the surgery instruments, to guide the tip inside the laparoscopic box and follow the motion commands from the user. The UR3 robots have a compact form making them suitable for this application with a small and tight workspace. The workspace of the laparoscopy instruments is limited by the laparoscopy training box and it corresponds to a space of approximately a 30 × 20 × 15 cm rectangular prism. The robot end-effectors controlling the instruments have a significantly larger workspace compared to this; therefore, all possible workspace within the training box and required for a typical suturing activity is reachable with the instrument tips. UR3 robot arms are collaborative robots with back drivable joints, which allows the user to guide the robot by hand and set the initial configuration with the laparoscopic instrument. The laparoscopic tool is inserted in the hole that simulates the incisions or the transabdominal working ports on patients. Another property of these collaborative robots is that they are safer compared to their industrial counterparts when hard collisions take place (Arents et al., 2021), but their repeatability precision is worse. However, the accuracy difference with industrial robot arms is not a critical factor for this application.

In order to sense contacts or collision at the forceps, a 6D Force-Torque sensor is attached to the wrist of the manipulators. This sensor allows estimating the direction and amount of force at the contact point at the tip of the instument.

**FIGURE 3:**
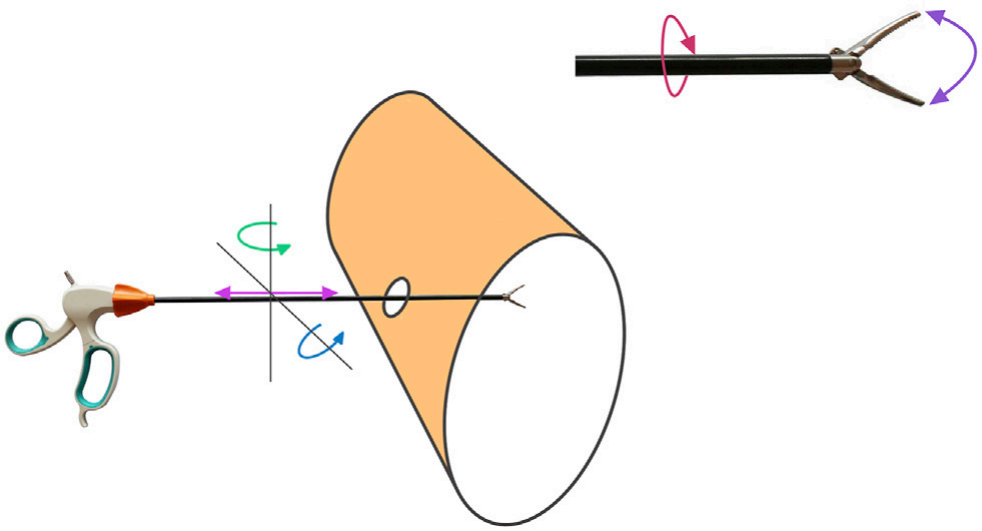
Actuated DOF per laparoscopic instrument.

**FIGURE 4:**
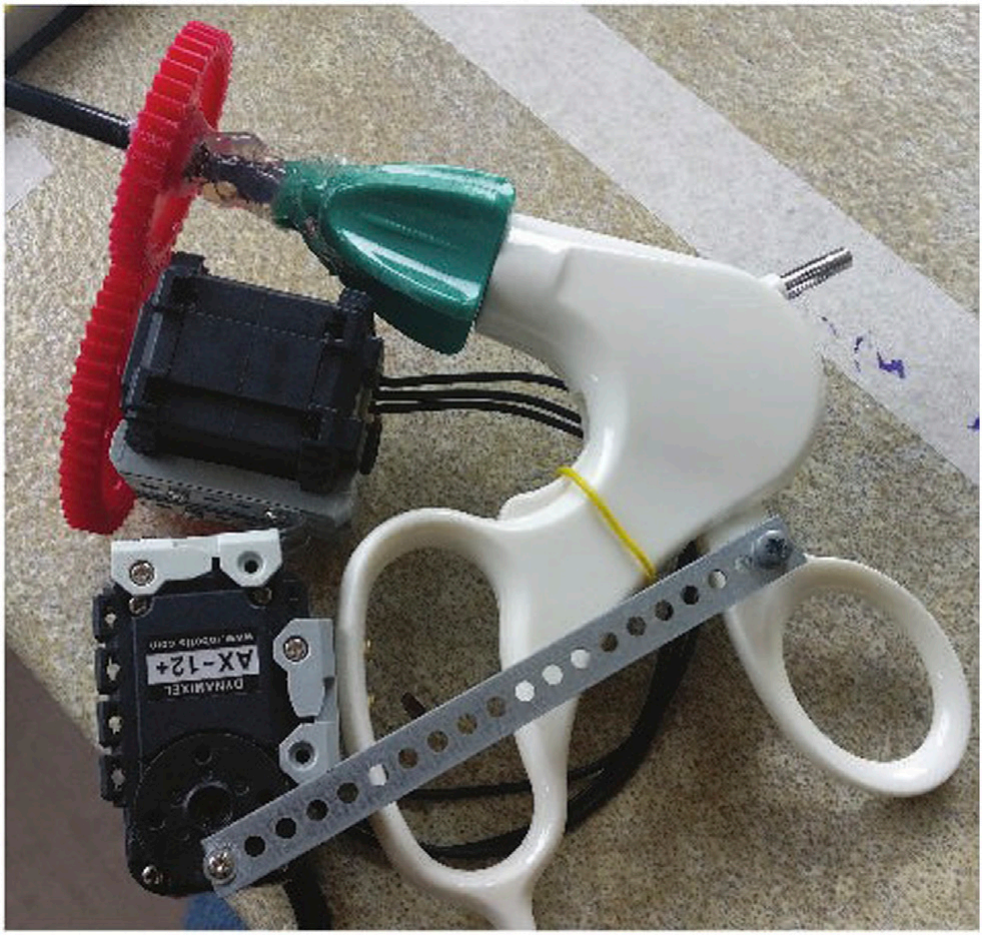
Two Dynamixel servos mounted on the forcep to control third rotational axis and grasping motion.

To have full control of the forceps we require to actuate on five degrees of freedom including the opening/closing of the forceps, for each instrument (see Figure 3). Three of those are controlled with the robot arm to change the position of the tip, and the other two are controlled with two servos that act on the laparoscopic instrument to control the rotation and opening/closing of the gripper (Figure 4). Considering that the robot has to move the forceps and follow the remote center-of-motion (RCM) constraint (defined by the hole in the box), we can only actuate 2 rotations and a translation with the robot arms. The translation is used to insert or extract the tool, and the two rotations control the position of the tip in the X-Z plane. The third rotation, the axial rotation of the forceps, is controlled not by the robot arm but by one of the servos attached to the customized forceps; and the opening/closing motion of the tool’s gripper is controlled by the second servo.

#### 2.1.2 Customized Forceps

A simple mechanism with two servos was designed to actuate on the two DOF that need to be actuated on the laparoscopic instruments (see Figure 4). One DOF, the axial rotation of the forceps, is continuous and without limits on rotation, while the grasp/release DOF of the gripper is continuous with limits (not binary). A Dynamixel smart servos is used to control each DOF. These servos were selected for their unlimited rotation and their capability to apply a position or torque control. The servos are interfaced with a serial protocol using an Arduino Mega controller.

The Dynamixel servos are connected in a daisy chain with serial communication using servo IDs to distinguish between the servos. Three serial ports are used for the slave side computer: one to send commands to the Arduino program and the other two for the Arduino to send commands to the Dynamixel servos (one serial port for each tool to communicate with). The Dynamixel AX-12 servos use a 3-Pin connector for ground, 9–12 V power, and data wires. The serial port for the Dynamixel servos can send and receive data along the single data wire and therefore requires bi-directional communication which is accomplished with the use of a tri-state buffer^6^.

The maximum force that can be applied to the objects in the scene is determined with setting a limit to the servo’s position. There is a maximum and minimum position defined in the Arduino code that stops the rotation of the servos if its position exceeds the set min/max. For example the closing/opening actuation stops when the gripper is either fully closed or opened. As a further development we note that setting a direct torque limit using the torque reading of the servos, would make the grabbing at the tool tip more adaptable to grasping objects with varying stiffness.

#### 2.1.3 Touch Haptic Devices

Two haptic devices are used to apply a bilateral tele-operation where motion commands are sent to the manipulators and force feedback is received when contacts are detected at the forceps. Each haptic device is composed of a 6 DOF serial kinematic chain where all the joints have position sensors but only the first three are motorized, which is sufficient for our purposes to indicate when a contact has taken place and to feedback the direction and amount of translational force interaction.

### 2.2 Tele-Operation

One of the major challenges of laparoscopic surgery is that surgeons have to compensate for the fulcrum effect. This means that if a surgeon needs to move the tool tip in the direction *x*, they need to move their hand in the direction of *-x* as the tool pivots around the incision. This is an obstacle that can be easily overcome in robotic surgery and therefore has also been compensated for in our system design.

For the tele-operation of the presented system, control with both the keyboard and the Touch haptic devices was implemented. While the keyboard offers a convenient solution that can be used remotely by anyone with access to a computer and internet, the Touch devices offer a more ergonomic, and intuitive interface for controlling the robots on-site. In both cases, the user can control five degrees of freedom: linear motion of the tool tip in three dimensions, rotation of the forceps, and grasping.

For the keyboard interface, the controls were based on the common “WASD” PC control scheme, whereas for the Touch devices the motion of the stylus was translated into the desired motion of the tool tip in the world frame. For the latter, a dead zone was implemented for both the linear and rotational motion of the stylus. This was done to ensure safety and to avoid moving the robot with any small, accidental motion of the hand. Motion commands are sent to the robot only when the user moves the stylus outside this dead zone. The buttons on the stylus of the haptic device were used to control the grasp and release motion of the tool. For the keyboard interface, in Table 1 the keyboard keys that can be used to control the left and right robotic surgical tools are outlined.

**TABLE 1:** Keyboard keys for tool teleoperation.

Tool tip motion	Keyboard keys (left)	Keyboard keys (right)
Up/Down	‘W’/‘S’	‘I’/‘K’
Left/Right	‘A’/‘D’	‘J’/‘L’
In/Out	‘E’/‘Q’	‘U’/‘O’
Rotate Clockwise/Counterclockwise	‘C’/‘X’	‘M’/‘N’
Grasp/release	‘R’/‘F’	‘Y’/‘H’

**FIGURE 5:**
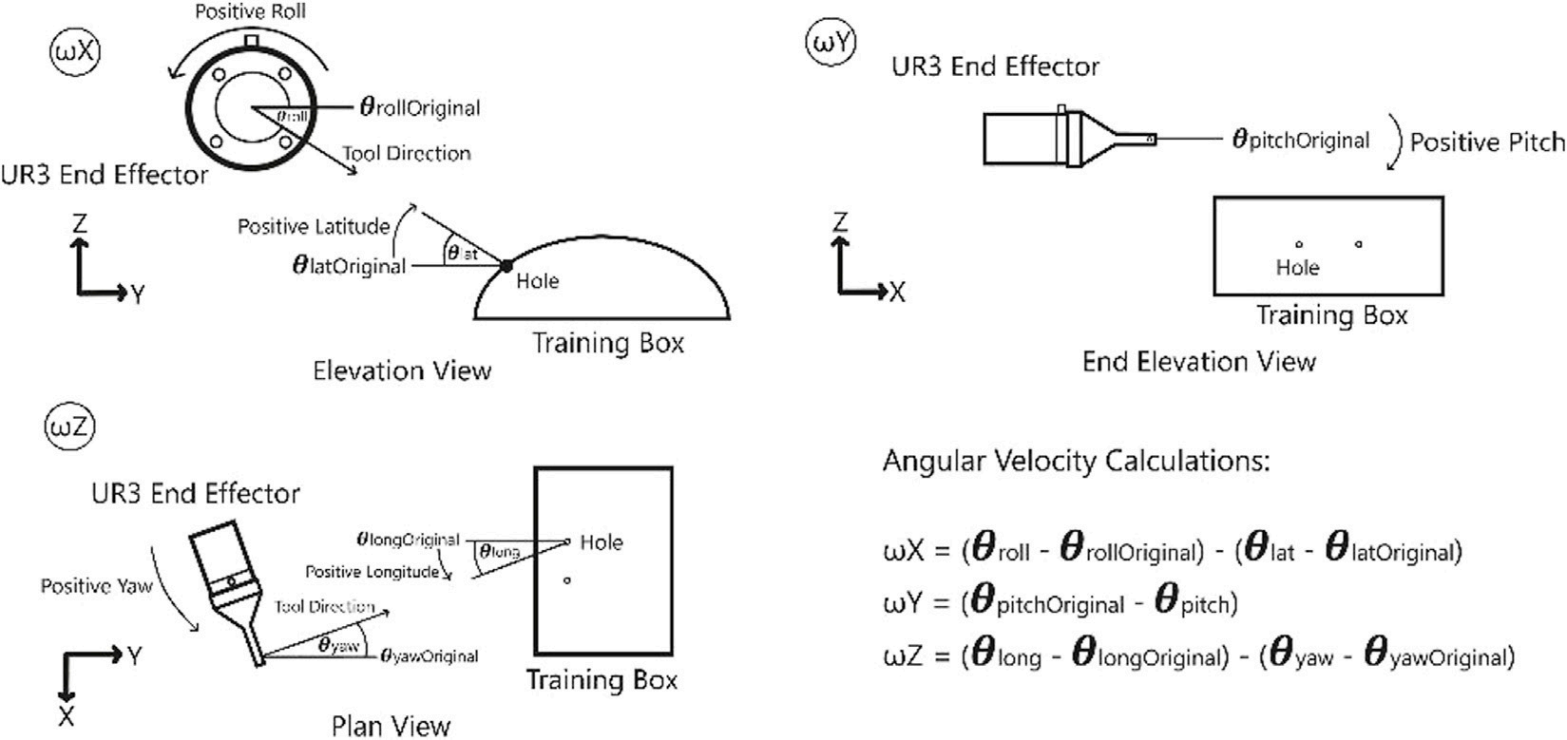
Coordinates used for the calculations of the linear and angular velocities of the robot.

In order to control the robot arms, the user commands are used to calculate the new position of the end effector of the robot. This is where the fulcrum effect is compensated for by the controller. For example, a “down” command by the user is translated as an upward motion of the robot in relation to its global frame. For the calculation of the end effector’s desired location, spherical coordinates are used. The position of the robot’s end effector, **P** ^*ee*^ = [*x* ^*ee*^, *y* ^*ee*^, *z* ^*ee*^], and the box’s hole, **P** ^*h*^ = [*x* ^*h*^, *y* ^*h*^, *z* ^*h*^] are used to calculate the radius, *r*, of the sphere, as well as the respective longitude, *ϕ*, and latitude, *θ* (Figure 5). With each user command, the desired position of the end effector is calculated by adjusting these parameters as follows:

$$\begin{array}{c} \theta_{i+1}=\theta_{i}+\delta \theta \\ \phi_{i+1}=\phi_{i}+\delta \phi \\ r_{i+1}=r_{i}+\delta r\\ x_{i+1}^{ee}=r_{i+1}cos\left(\theta_{i+1}\right)sin\left(\phi_{i+1}\right)+x^{h}\\ y_{i+1}^{ee}=r_{i+1}cos\left(\theta_{i+1}\right)cos\left(\phi_{i+1}\right)+y^{h}\\ z_{i+1}^{ee}=r_{i+1}sin\left(\theta \right)+z^{h} \end{array}$$

where |*δθ*| = |*δϕ*|, *δr* = 0 unless an in/out command is received, and sin(*ϕ*) = 1 and sin(*θ*) = 1 when the “left” and “down” commands are received, respectively, either from the stylus of the haptic device or from the keyboard of the remotely controlling computer.

A unit vector which points from $P_{i}^{ee}$ to $P_{i+1}^{ee}$ is then constructed and is used to define the linear velocity of the robot’s end effector. To satisfy the motion constraint imposed by the hole, the rotational velocities of the robot’s end effector are then calculated to ensure that any change in the longitude or the latitude of the system generates the equivalent change in the orientation of the end effector of the robot. The starting orientation of the robot is saved, **O**_0_ = [*roll*_0_, *pitch*_0_, *yaw*_0_] along with the initial longitude, *ϕ*_0_, and latitude, *θ*_0_, of the system and the rotational velocities are calculated as:

$$\begin{array}{c} \omega_{X}=\left(\theta_{i+1}-\theta_{0}\right)-\left(roll_{i}-roll_{0}\right)\\ \omega_{Y}=\left(pitch_{0}-pitch_{i}\right)\\ \omega_{Z}=\left(\phi_{i+1}-\phi_{0}\right)-\left(yaw_{i}-yaw_{0}\right) \end{array}$$

### 2.3 Haptic Feedback

A major advantage of using a real robotic surgery training setup compared to a simulator is that realistic haptic feedback can be provided. Studies about the usefulness of haptic feedback for training show that “force parameters and force feedback in box trainers improve training results”, Overtoom et al. (2019). The force feedback can help the trainees to get a better “feeling” on how much force they exert on the environment, which is a crucial learning goal for robotic surgery performance and patient safety. They can learn to cognitively connect the force feedback that they experience to the visual effects on the objects they manipulate.

**FIGURE 6:**
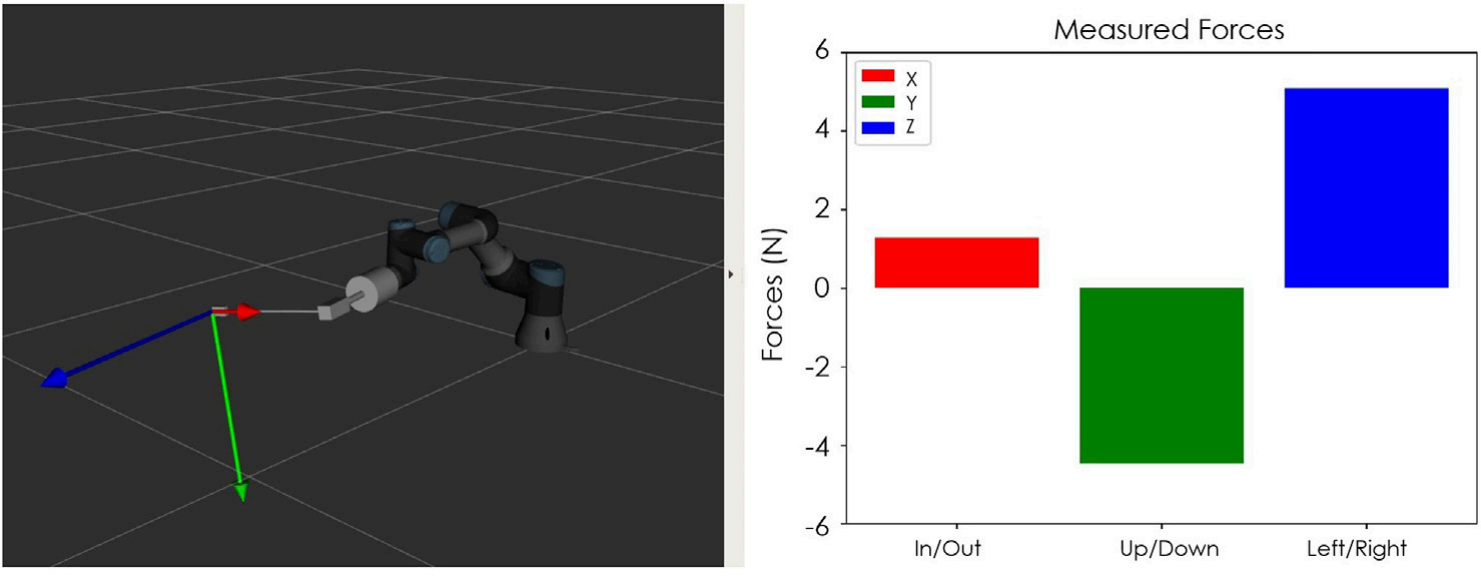
Visual feedback of forces that can be provided during the teleoperation of the robot by keyboard.

With the use of the force-torque sensors attached at the end effector of each robot, the interaction forces between the robot and the environment can be measured and can be reproduced by the Touch devices to provide haptic feedback to the user. The force and torque data measured at the sensor not only include the force data resulting from contacts at the interaction port, but also the gravitational and inertial components from the tool and the laparoscopic instrument. Our system eliminates the gravitational components in force sensing by simply setting the force-sensor reading to zero when there is no contact at the instrument tip. As the orientation of the instruments do not change much during laparoscopy exercises, elimiation of the gravitational forces at the start of the operation is considered to be satisfactory for our system. Besides that the tools move very slowly during robotic training, which implies that the inertial forces resulting form the movements are almost zero at all times. Therefore, simply eliminating the gravitational forces at the start of the operation has been observed to be effective to register reliable force sensor signals to generate meaningful haptic feedback to give a good sense of the interaction forces, as we demonstrate below with a sample set of recorded force data in the interaction and feedback side. With three actuated degrees of freedom, the Touch devices can provide force feedback in three dimensions in the Cartesian space. This will allow the user to sense if they are pulling or pushing against a surface and thus move the robot accordingly as desired. While this can be achieved with the haptic devices, we note that there are still a couple of degrees of freedom that can not be interfaced with the force feedback in the haptic device, and the rotation of the tool and the gripper force. When a user operates the robots through the keyboard, the sensed forces can be provided visually in the form of an animation accompanied by a graph on the screen (Figure 6).

**FIGURE 7:**
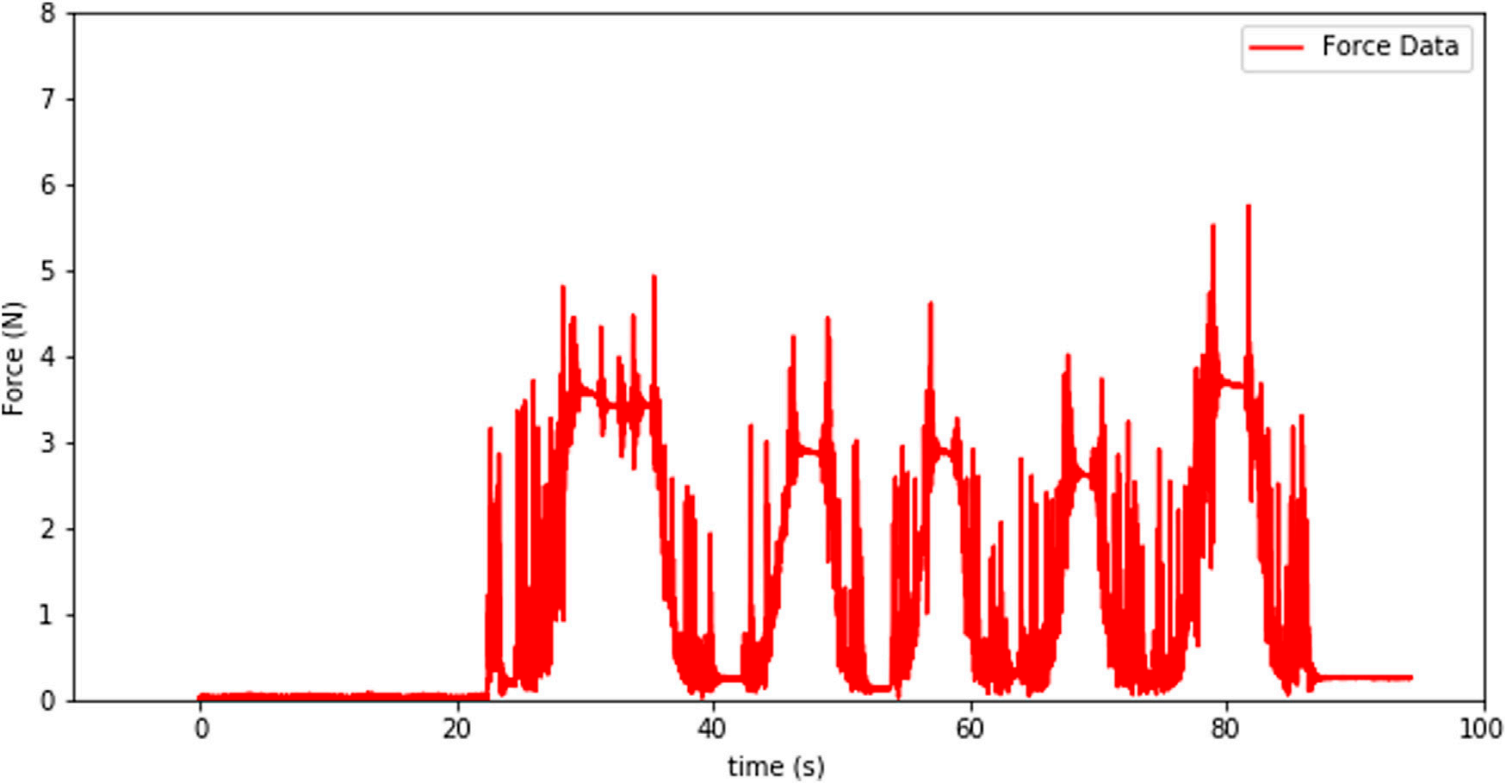
Instrument tip force computed based on the force-torque sensor signal.

**FIGURE 8:**
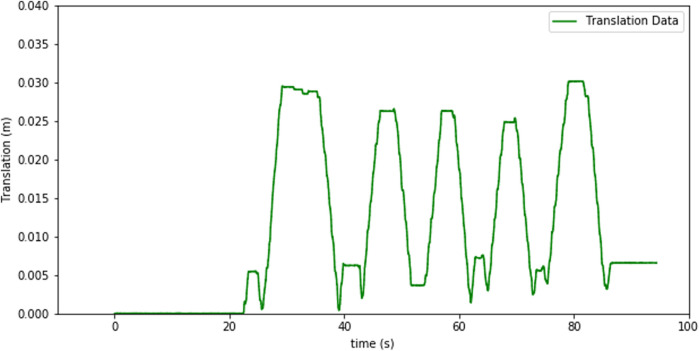
Tool-tip position over time.

**FIGURE 9:**
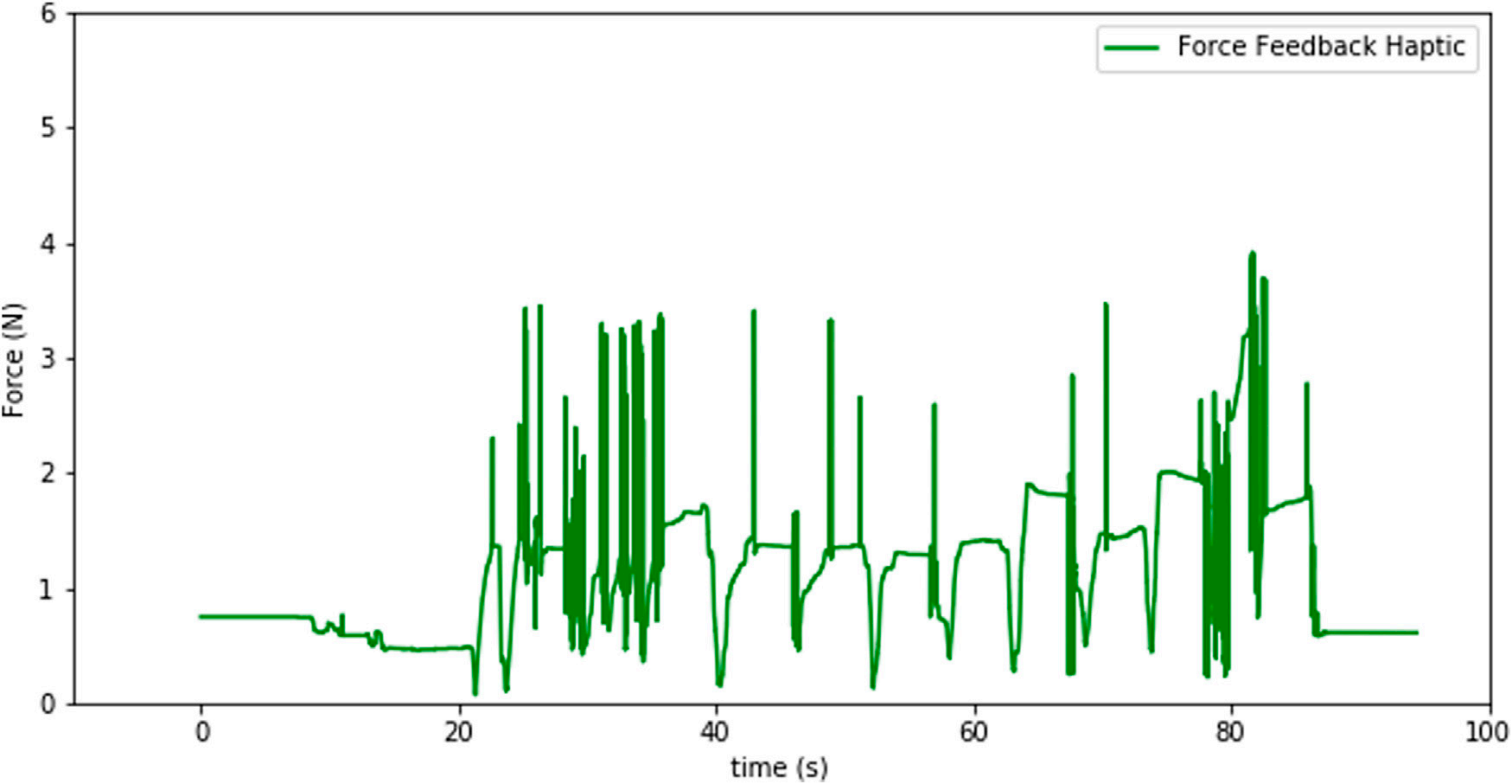
Force feedback sensation at the haptic device.

The signal from the force-torque sensor mounted on the wrist of the robot is first used to compute the estimated force at the tip of the instrument and then this force value is sent to the haptic device to give the user haptic feedback. The haptic feedback is implemented to give the trainee a “sense of touch” while completing the training tasks. The graphs in Figures 7–9 respectively show a sample recording of the instrument tip force computed based on the force-torque sensor signal, the position of the tool tip, and the force feedback signal the user receives through the haptic device.

The spikes in the tip-point force data and also some in the haptic sensation plot (see Figures 8, 9) occur due to three factors: at the instants that a contact is established, structural flexibility of the laparoscopy instrument, and some sensor noise attenuated due to those two impacts. The haptic device was used to send motion commands to the robot while receiving the force feedback from the sensor (see Figure 7). For this experiment a foam material was used and the tool tip was pressed five times into the material as can be seen in the change of the tool-tip position in Figure 8.

The coordinate frame from the sensor differs from the coordinate frame of the tool-tip. Therefore, we need to compute the forces/torques at the tool-tip using the forces/torques at the sensor in the wrist. For this calculation we used the formula in Eq. 1 which makes use of the rotation matrix between the coordinate frames {A} and {B} and the position vector between the origins of these two frames, Hu (2001):

$$\begin{array}{l} {\left[\begin{array}{c} f_{tx}\\ f_{ty}\\ f_{tz}\\ \tau_{tx}\\ \tau_{ty}\\ \tau_{tz} \end{array}\right]}^{B}=\left[\begin{array}{cc} R_{ba} & 0\\ S\left(P\right)R_{ab} & R_{ba} \end{array}\right]{\left[\begin{array}{c} f_{sx}\\ f_{sy}\\ f_{sz}\\ \tau_{sx}\\ \tau_{sy}\\ \tau_{sz} \end{array}\right]}^{A} \end{array}$$ (1)

$$\left\{A\right\}:\text{coordinate frame of the force sensor}$$

$$\left\{B\right\}:\text{coordinate frame of the tool}-\text{tip}$$

$$R_{ba}:3\text{x}3\text{ rotation matrix from frame}\left\{B\right\}to\left\{A\right\}$$

$$\left[\begin{array}{ccc} f_{tx} & f_{ty} & f_{tz} \end{array}\right]:\text{Forces acting on the tool}-\text{tip in x, y, z directions of reference frame}\left\{B\right\}$$

$$\left[\begin{array}{ccc} f_{sx} & f_{sy} & f_{sz} \end{array}\right]:\text{Forces acting on the sensor in x, y, z directions of reference frame}\left\{A\right\}$$

$$\left[\begin{array}{ccc} \tau_{tx} & \tau_{ty} & \tau_{tz} \end{array}\right]:\text{Torques acting on the tool}-\text{tip in x, y, z directions of reference frame}\left\{B\right\}$$

$$\left[\begin{array}{ccc} \tau_{sx} & \tau_{sy} & \tau_{sz} \end{array}\right]:\text{Torques acting on the sensor in x, y, z directions of reference frame}\left\{A\right\}$$

$$P_{ba}=\left[\begin{array}{ccc} p_{x} & p_{y} & p_{z} \end{array}\right]:3\text{x}1\text{ position vector of the origin of A coordinate frame in B coordinate frame}$$

$$S\left(p\right):\text{is a }3\times 3\text{ skew matrix of the position vector P }$$

$$\begin{array}{l} {\left[\begin{array}{c} f_{tx}\\ f_{ty}\\ f_{tz}\\ \tau_{tx}\\ \tau_{ty}\\ \tau_{tz} \end{array}\right]}^{B}=\left[\begin{array}{cc} I & 0\\ S\left(P\right)I & I \end{array}\right]{\left[\begin{array}{c} f_{sx}\\ f_{sy}\\ f_{sz}\\ \tau_{sx}\\ \tau_{sy}\\ \tau_{sz} \end{array}\right]}^{A} \end{array}$$ (2)

I:3x3 identity matrix

**FIGURE 10:**
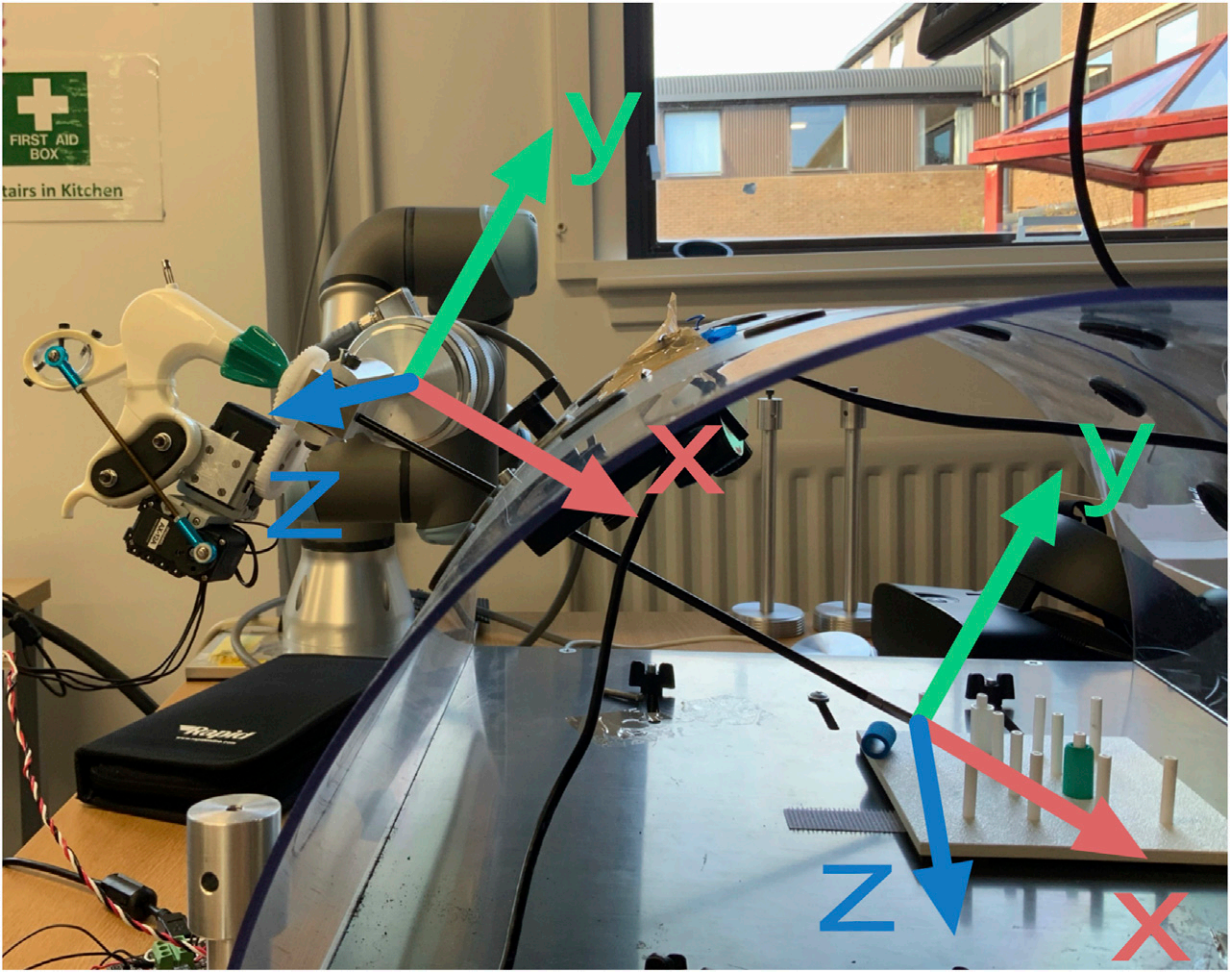
Coordinate frame conversion from force torque sensor attached to the wrist of the robot arm to the tool-tip coordinate frame.

As can be seen in Figure 10 the transformation between the two frames only consists of translational difference in the form of the position vector from one frame origin to the other. There is no rotation transformation from frame {A} to frame {B}, which means that the rotation matrix in Eq. 1 is the identity matrix. The calculated forces in Eq. 2 are sent to the haptic device to generate the force feedback provided to the user.

### 2.4 3D Vision

**FIGURE 11:**
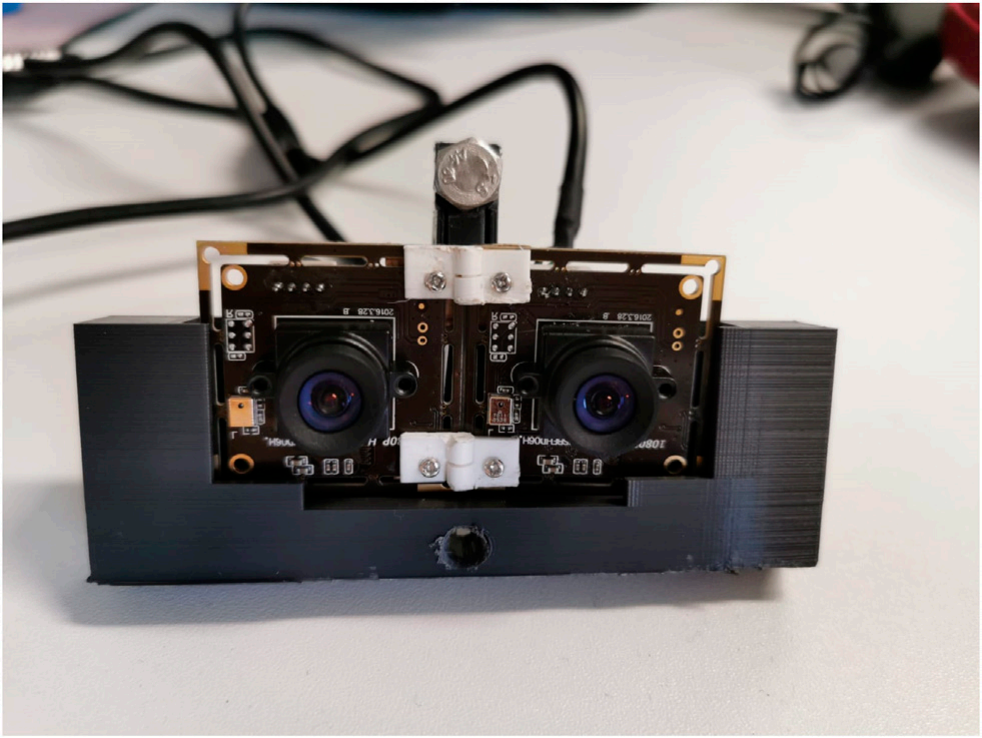
Stereo vision setup with two wide-angle cameras in a 3D printed mount, put in right position and orientation by 3D printed hinges.

The goal of a training system is to mimic the real system or process trainees are training for, as closely as possible. Since in some robotic surgery systems 3D or stereo vision is available to the surgeon, this project aimed for finding a low-cost component to introduce 3D vision to the training system as well. The hardware we used to accomplish the implementation of 3D vision in the training system is an Oculus Rift S VR headset, two wide-angle cameras (ELP Sony IMX322 Sensor Mini USB Camera Module HD 1080P; None Distortion 100 Degree), and a 3D printed mount that was designed to fit the cameras and the training box frame (Figure 11).

One of the challenges in implementation of 3D vision is to find the right orientation and positioning of the cameras in relation to each other. We solved this by printing customized hinges and trying out several camera mount designs. To get a correct stereo vision, the right interpupillary distance (IPD) must be found. IPD is defined by Shafiee et al. (2014) as the “distance between the centres of the pupils”. It determines the angle of disparity between the two observed images which are combined in the brain of the observer to produce stereo perception. For our 3D system the two camera streams received from the operation space inside the laparoscopy box are fed into the Oculus Rift S headset, one video stream for each eye. To get this graphical representation, OpenGL and OpenCV were integrated into the OculusRoomTinyGL.vcxproj which can be found as a part of the SDK of Oculus Rift (https://developer.oculus.com/downloads/package/oculus-sdk-for-windows/).

### 2.5 Remote Control

The main aim of the remote tele-operation for this robotic surgery training system is to enable a wide spread access to the system through internet, without implying installation and transportation costs. In this way, and in principle, anyone with access to internet can physically manipulate the robotic training system and acquire a feeling of how tele-manipulation of a surgical robot works. The tele-operation is developed such that the user can control the training system from their computer by sending commands over the internet while receiving a video stream of the training site. The key requirement for the successful implementation of this system is to accurately transmit control commands while receiving video with low latency.

#### 2.5.1 System Architecture

To allow for access over the internet a Client-Server architecture was chosen. The main advantage of this architecture is that it allows for having distinct workloads between computers. In this case, three computers are needed. Two of the computers act as clients by sending and receiving control and video streams. The third computer acts as a server with a static IP for both clients to create a connection.

**FIGURE 12:**
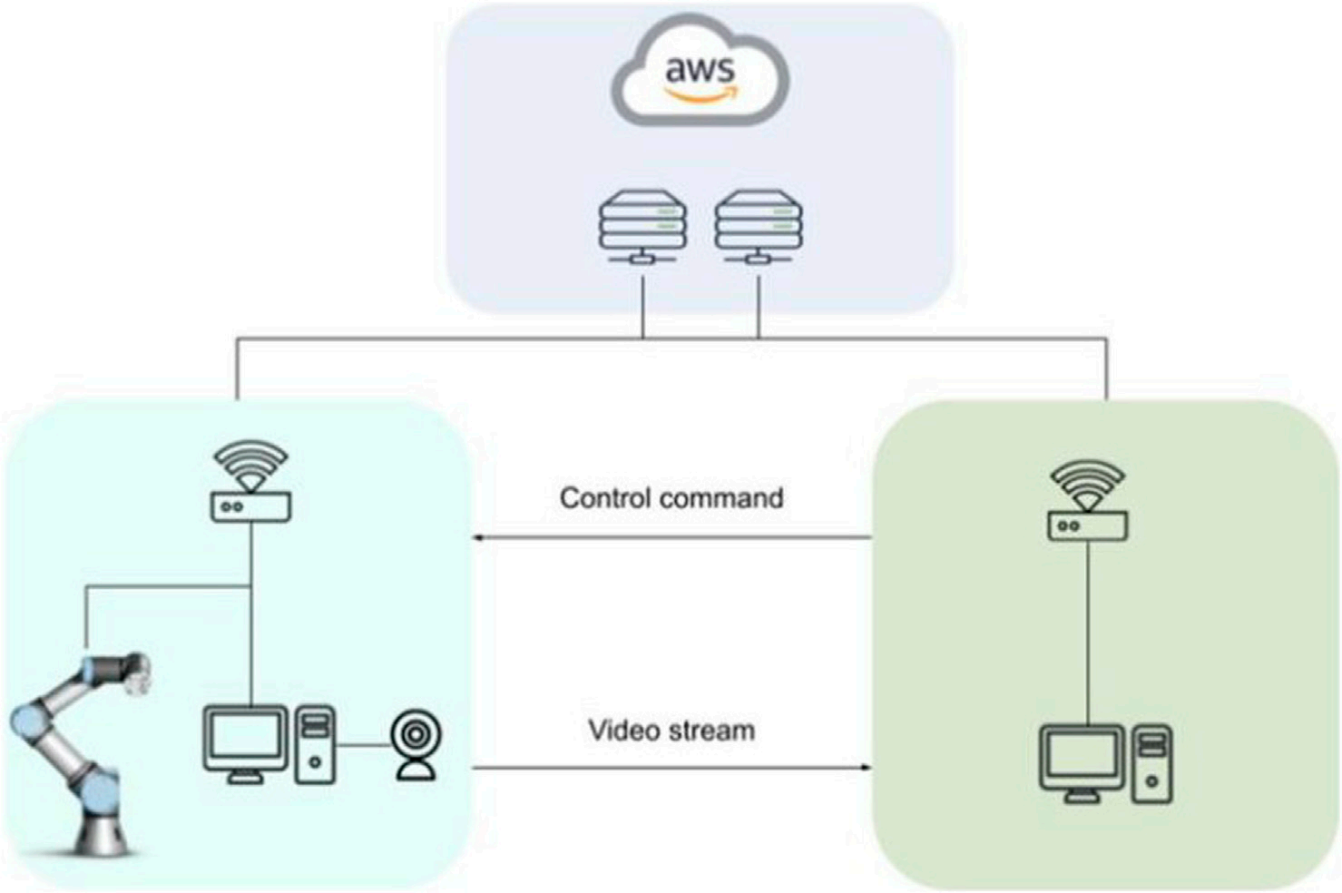
Client-Server architecture with cloud servers for remote control of the robotic training setup.

The overall architecture of the system is shown in Figure 12. In this architecture, both the robot and the user act as clients and the servers which transmit data back and forth deployed on a virtual machine in instances hosted on Amazon Web Service’s EC2. The primary reason for choosing Amazon Web Service was the ability to have access to the servers through public static IPs from white-listed machines. Additionally, to separate the flow of data streams, two separate servers were used one for the video stream and another for control commands.

#### 2.5.2 Communication Protocol

The Transmission Control Protocol (TCP) was chosen as the communication model between the clients and the servers. The key reason for using TCP was the requirement for reliable transmission of the data due to the potential variability in the latency as the requirement for accurate transmission. The ability of TCP to retransmit and detect errors improves the reliability of the communication although at the cost of some latency.

#### 2.5.3 Compression and Performance

The accurate control of the setup is dependent on two separate processes. First, the command data from the controller, for example a keyboard, and should be transmitted with low latency. However, more importantly, the visual feedback is a key component to successful teleoperation and as a result, fast and high-quality video transmission is required. The fast transmission of the video feed is a complex task due to the large packet size and the delicate balance between quality and latency. For this project, the objective is to reduce the latency as much as possible by compressing the packet size, while maintaining the required amount of information transfer to receive accurate visual feedback. Table 2 presents the results of the trade-off between resolution and compression and their effect on latency in the form of frames per second (FPS) and latency in milliseconds.

**TABLE 2:** Effect of JPEG compression on latency and FPS.

Compression %	1080 × 920		720 × 480	
	FPS	Transmission time (ms)	FPS	Transmission time (ms)
90	31	31.1	31	30.0
70	30	35.2	31	30.8
50	29	35.6	30	31.1
30	24	24.5	30	31.4
10	12	75.8	29	32.6
0	4	280.2	12	70.3
Average	21.6	482.4	27.2	37.7

From the table, it is clear that the reduction in resolution in this case from 1080 × 920 to 720 × 480 leads on average to a better frame transmission at 27 FPS (720 × 480) compared to 22 FPS (1080 × 920). This is a 22.7% improvement. Additionally, we compare the effects of JPEG compression on the FPS. The results show that in the case of higher resolution (1080 × 920), compression can be reduced to 50% with minimal effect on the FPS. While the lower compression leads to noticeably worse performance. Concerning the lower resolution, the effect is similar however the drop in FPS is observed at much lower compression rates.

Therefore based on the results above, we chose using the lower resolution of 720 × 480 with 50% compression, since the information loss was minimal and latency was low enough for the user commands to be in synchrony with the visual feedback.

#### 2.5.4 Latency

**FIGURE 13:**
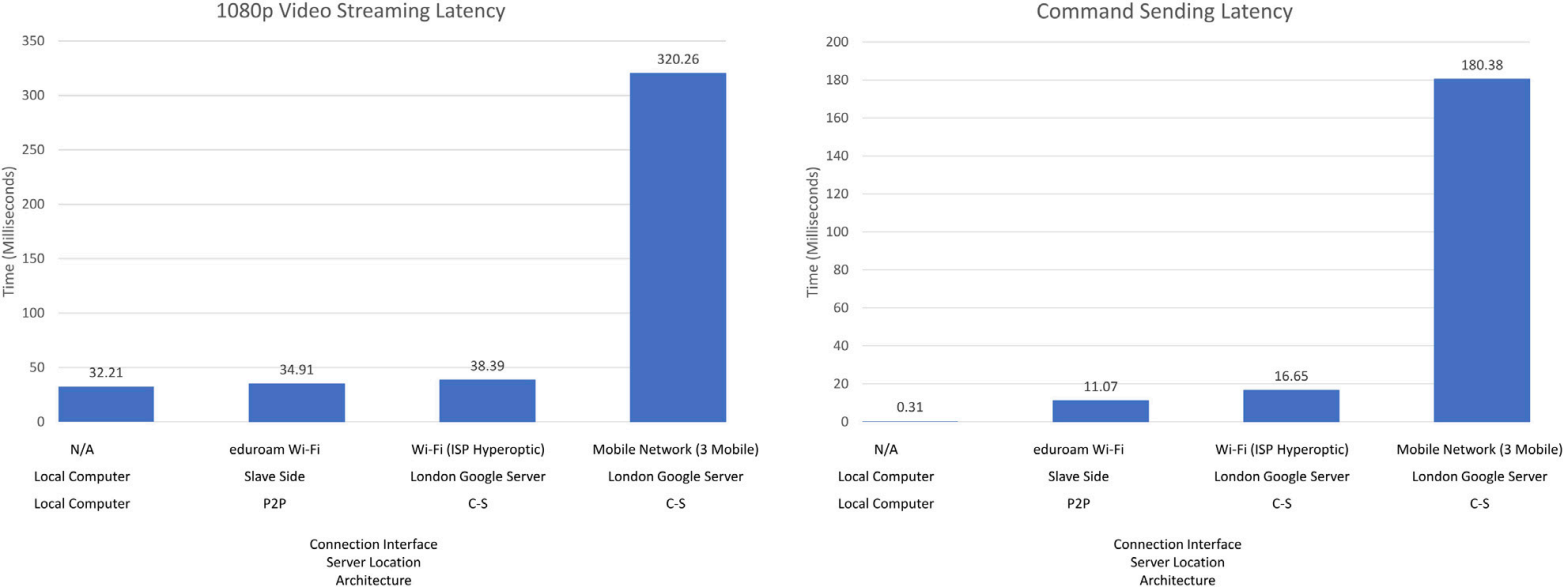
Command sending latency (left) and Video Streaming latency (right).

The latency is most critical considering the remote use of the training setup through internet connection. This latency is dependent on the performance and speed of the commands and video transmission. We measured the latency in four different situations (Figure 13). The first one was when running the server, master and slave programs on the same computer, while those programs being connected to the localhost. The second one was the realistic testing situation under the Wi-Fi network of Heriot Watt University (100 Mbps), where the devices get a public IP address under this network, and the server programs were running on the slave side computer for establishing P2P architecture. The third one was while connecting both master and slave sides to the Google server located in London, while the Wi-Fi was powered by Hyperopic (150 Mbps) which does not provide a static and public IP address to the users. The fourth one was while connecting to the Google server with a 4G mobile network powered by 3 United Kingdom (11.45 Mbps).

The first situation represented by the left-most bars implies the lowest latency. The first situation, namely the local connection, did not transfer any data through the Internet, therefore, the latency result of this situation make sense only as a reference for the fastest possible transmission that the programs can perform. The performance of both the second and third situations were in an acceptable range; because of the connection distance, the second situation was slightly better than the third one. However, the experience also showed that the performance varied due to network quality and speed. The same setup was used in the third and fourth situations, the only difference being the network connection interface. With Hyperopic Wi-Fi, command sending and video streaming worked 10 times faster than the 3 United Kingdom 4G mobile network, obviously verifying that the quality and speed of the network is a critical factor for data transmission through the Internet. These results indicate that the command sending and compressed video streaming we have implemented perform very good (with less than 50 ms delay) under the first three plausible network settings or architectures and satisfactorily (with less than 350 ms delay) with the Mobile Network. While a slower speed network might cause higher latency for both sets of programs, with the 10 Mbps network, the latency was 1/5 s for command sending and 3/10 s for video sending with the Mobile Network. Therefore, the training system as it is now should be used with a reliable high-quality network environment, such as universities, hospitals or government buildings.

## 3 Discussion and Future Work

The Robotic Surgery Training System presented in this paper can be used remotely by anyone who has access to a personal computer with connection to the internet or on-site with either the keyboard controls or Touch haptic devices. Whether on-site or remotely, visual feedback can be provided on the computer’s screen to allow the user to observe the tool’s motion inside the training box as well as the magnitude and direction of the forces that are computed for the instrument tip-point interaction, based on the force signal registered by the force sensors that are placed at the end-effector of the robots. On-site training also allows for haptic feedback to be provided when the Touch haptic devices are used. The system also provides 3D visual feedback with the Oculus Rift headset.

Considering the remote use of the training system, one of the major obstacles for a user-friendly control is the effect of latency in data transmission, specifically when it comes to the video stream. As explained in Section 2.5.3 the user heavily relies on the video transmission for controlling the robot. In the current state of the system, to reduce the latency, the resolution of the feed was lowered, and the transmitted information was compressed to 50% by using JPEG compression. However other compression formats are also available which outperform JPEG and are dedicated to video encoding. For example, H264 is a dedicated video coding method. A great number of empirical data shows that H264 greatly outperforms JPEG by using significantly less bandwidth, as a result allowing for much faster transmission of data Lian et al. (2005). This is a future work that we consider implementing in our system to speed up data transmission for remote control.

Another limitation of the current setup is that force feedback for grasping cannot be provided. Even though the tools have been customized to gain control over the tool’s rotation and grasping capabilities, a force sensor is not available at the tip of the tools, and at the moment. The smart servos that are used in the laparoscopic instrument have the capability to return load feedback, so this could be a way to estimate those forces. However, an actuator that will provide this force feedback to the user is currently absent. This is something that can be added to the system in the future using additional actuators such as wearable fingertip actuators, as the ability to sense the grasping forces that the tools apply to their environment is crucial to ensure that the soft materials to be manipulated are not damaged.

Future studies can investigate the system’s ability to improve the user’s performance when performing a task with augmented visual and/or haptic feedback. It is also of interest to study the long-term effects of training with such a setup and observe/quantify the transferable skills to a real robotic-surgery system.

## Data Availability

The original contributions presented in the study are included in the article/Supplementary Material, further inquiries can be directed to the corresponding author.

## Footnotes

1
https://www.3dsystems.com/haptics-devices/touch.

2
http://simulatedsurgicals.com/projects/ross/.

3
https://mimicsimulation.com/dv-trainer/.

4
https://www.davincisurgery.com/da-vinci-systems/about-da-vinci-systems.

5
https://www.scientificamerican.com/article/robotic-surgery-opens-up/.

6
https://robottini.altervista.org/dynamixel-ax-12a-and-arduino-how-to-use-the-serial-port?doing_wp_cron=1633 432 382.438 378 095 626 831 054 687 5.
